# Electrocomposite Developed with Chitosan and Ionic Liquids Using Screen-Printed Carbon Electrodes Useful to Detect Rutin in Tropical Fruits

**DOI:** 10.3390/s18092934

**Published:** 2018-09-04

**Authors:** Lili Muñoz, Verónica Arancibia, Olimpo García-Beltrán, Edgar Nagles, John J. Hurtado

**Affiliations:** 1 Ingeniería Agroindustrial, Facultad de Ingeniería Agronómica, Universidad del Tolima, Calle 67 No. 53-108 B, Ibagué-Tolima 730001, Colombia; lilifasulimondragon@gmail.com; 2 Facultad de Química, Pontificia Universidad Católica de Chile, Vicuña Mackenna 4860, Santiago 7820436, Chile; darancim@uc.cl; 3 Facultad de Ciencias Naturales y Matemáticas, Universidad de Ibagué, Carrera 22 Calle 67, Ibagué 730001, Colombia; jose.garcia@unibague.edu.co; 4 Departamento de Química, Universidad de los Andes, Carrera 1 No. 18A-12, Bogotá 111711, Colombia; jj.hurtado@uniandes.edu.co

**Keywords:** adsorptive voltammetry, rutin, ionic liquids, chitosan, tropical fruits

## Abstract

This work reports the development of a composite of the ionic liquid 1-butyl-3-methylimidazolium tetrafluoroborate ([BMIM]BP_4_) and chitosan (CS) described in previous reports through a new method using cyclic voltammetry with 10 cycles at a scan rate of 50.0 mV s^−1^. This method is different from usual methods such as casting, deposition, and constant potential, and it allows the development of an electroactive surface toward the oxidation of rutin by stripping voltammetry applied to the detection in tropical fruits such as orange, lemon, and agraz (*Vaccinium meridionale* Swartz), with results similar to those reported in previous studies. In addition, the surface was characterized by electrochemical impedance spectroscopy (EIS), scanning electron microscopy (SEM), and Raman spectroscopy. The limit of detection was 0.07 µmol L^−1^ and the relative standard deviation (RSD) of 10 measurements using the same modified electrode was 0.86%. Moreover, the stability of the sensor was studied for six days using the same modified electrode, where the variation of the signal using a known concentration of rutin (RT) was found to be less than 5.0%. The method was validated using a urine chemistry control spiked with known amounts of RT and possible interference was studied using ten substances including organic and biological compounds, metal ions, and dyes. The results obtained in this study demonstrated that this electrodeveloped composite was sensitive, selective, and stable.

## 1. Introduction

In the last decade, ionic liquids (ILs) and chitosan (CS) have been widely used in the modification of electrodes used as electrochemical sensors. This has been mainly due to the advantageous properties of CS such as good biocompatibility, biodegradability, and adhesion [1] that allow the immobilization of enzymes [2] and single stranded deoxyribonucleic acid (ssDNA) [3] through the charge interaction at acidic pH values, and properties of ILs such as high ionic conductivity, high viscosity, ionic structure, and low volatility [4,5]. Moreover, imidazolium and pyridinium groups are the most used cations, and the hexafluorophosphate and tetrafluoroborate ions are the most used anions in electrode modification [6]. Therefore, they have been used in the detection of biological substances such as dopamine and uric acid [7,8,9], organic substances such as hydroquinone [10], fephol [11], and hydrazine [12], fumigants such as imidacloprid [13,14], toxic metal ions such as Pb and Cd [15,16], and for microextraction of pesticides [17].

The combination of CS with ILs was studied by Chen et al., who reported that the imidazolium cation is dissolved more effectively with chitosan through hydrogen bonds [18]. Moreover, this combination has been used for CO_2_ capture [19]. Therefore, this composite has been used in the electroanalytical detection of formaldehyde and nitrite with 1-octyl-3-methylimidazolium hexafluorophosphate, [OMIM]PF_6_ [20,21], cholesterol with 1-butyl-3-methylimidazolium chloride, [BMIM]Cl [22], theophylline with 1-(3-Aminopropyl)-3-methylimidazolium bromide [AMIM]Br [23], glucose [24], hydrogen peroxide and luteolin with 1-butyl-3-methyl-imidazolium hexafluorophosphate [BMIM]PF_6_ [25,26], and ssDNA with *N*-hexylpyridinium hexafluorophosphate [HPP]F_6_, and 1-butylpyridinium hexafluorophosphate [BPP]F_6_ [27,28]. Additionally, this composite has been used as a catalyst in the Heck reaction of aryl bromides [29] and in the development of the membranes for the fabrication of dye-sensitized solar cells [30].

In particular, the composite formed between CS and [BMIM]PB_4_ is one of the most reported in the development of electroanalytical sensors. It has been used to detect trichloroacetic acid [31], hydrogen peroxide [32,33,34], glucose [35], NADH [36], cholesterol [37], guanine and adenine [38], and bisphenol [39]. In these studies, glassy carbon, carbon paste, and gold were the most commonly used electrodes. On the other hand, the CS and IL properties were mainly controlled by casting, deposition, and the substrate, and less often by electrodepositing at a controlled potential.

Interest in detecting rutin (RT) or vitamin P in natural products such as fruits, coffee, and tea is motivated mainly by their potential activity, such as antioxidant, anti-inflammatory, blood-vessel-protecting and anticancer-agent activities [40,41]. The detection of RT using CS in electrode modification has been reported with multiwalled carbon nanotubes [42,43], graphene [44] and graphene with poly (amido amine) [45]. Moreover, the use of ionic liquids to detect rutin has also been reported using pyridinium and imidazolium ionic liquid on a modified carbon paste and glassy carbon electrodes with detection limits between 0.6 nmol L^−1^ and 0.09 μmol L^−1^ [33,46,47,48,49,50,51,52,53]. On the other hand, CS and IL composites with rutin have been recently reported to act as an immunosensor with multiwalled carbon nanotubes and [BMIM]BF_4_ [54]. Most of these reports use other substances such as graphene, carbon nanotubes, nanoparticles, and conductive polymers. By contrast, there have been no reports of the use of screen-printed carbon electrodes that are very versatile for this type of study.

The previous studies described above demonstrate the importance of detecting RT and the versatility of chitosan and ionic liquids for modifying the electrodes.

In this study, we evaluated the electrochemical oxidation of rutin using a screen-printed carbon (SPC) electrode coated with a CS-IL composite. The development of a simple method that allows the detection of RT in tropical fruits is the aim of this work.

## 2. Materials and Methods

### 2.1. Apparatus

Cyclic voltammograms (CVs) and square wave-stripping voltammograms (SWAdVs) were obtained using a DropSens µStat400 potentiostat (Oviedo, Spain). Electrochemical impedance spectroscopy (EIS) was carried using a VersaSTAT 3 Potentiostat/galvanostat from Princeton Applied Research (Oak Ridge, TN, USA) Scanning electron microscopy (SEM) was performed using a JEOL, model JSM 6490-LV (Tokyo, Japan) with a secondary electron detector. Raman spectroscopy was performed with a RIBA Yovin-Ivon spectrometer (Kyoto, Japan) using various laser wavelengths (532, 638 and 786 nm). The electrochemical cell consisted of a screen-printed carbon electrode (DRP C110. DropSens, Oviedo, Spain) with carbon as the working electrode (4 mm), Ag as the reference electrode, and carbon as the counter electrode. The pH measurements were carried out using an Orion-430 digital pH/mV meter equipped with a combined pH glass electrode.

### 2.2. Chemicals and Reagents

Ultrapure water was obtained using a Wasselab Purifier System. Methanol, phosphoric acid, NaH_2_PO_4_, and Na_2_HPO_4_ were obtained from Merck (Darmstadt, Germany). Chitosan (low molecular weight), 1-butil-3-metilimidazolio tetrafluoroborate, and K_4_Fe(CN)_6_ were obtained from Sigma–Aldrich (Milwaukee, WI, USA). The stock solutions, rutin, morin, quercetin, dopamine, ascorbic acid, uric acid, hydroquinone, tartrazine, and sunset yellow (Sigma–Aldrich) were prepared only once for the entire study (0.6 mmol L^−1^) in methanol. The phosphate buffer solutions (PBS) as electrolyte were prepared in the pH range between 2.0 and 7.0 range using 0.010 mol L^−1^ phosphoric acid, sodium phosphate, and disodium phosphate solutions.

### 2.3. Preparation of Electromodified SPC Electrode with Chitosan and Ionic Liquids (IL-CS/SPC)

The SPC electrode surface was washed with ultra-pure water. The electromodified IL-CS/SPC electrode was prepared as follows: first, the composite of CS and ILs was prepared using 100.0 µL of CS optimal solution (between 2.5 and 13.5 mg of CS in 10.0 mL acetic acid 1.0%) and 100.0 µL of pure ILs. Then, 20 µL of the freshly prepared solution was added on the surface of the SPC electrode. Then, the IL-CS/SPC-modified electrode was subjected to 10 cycles between –0.3 and 1.0 V at a rate of 0.05 Vs^−1^ and was washed with ultrapure water to remove the excess solvent.

### 2.4. Samples Preparation

The orange, lemon, and agraz tropical fruits were obtained from a supermarket in Ibague, Colombia. The extraction was carried with a juice extractor via a conventional method. Agraz (*Vaccinium meridionale* Swartz) was dried at room temperature and crushed in a disc mill. A total of 500 g was extracted diligently in methanol in a 5 L vessel for 15 days and shaken daily. The obtained extract was concentrated under reduced pressure in a vacuum brand system. No pretreatment was necessary before each analysis.

### 2.5. Measurement Procedure

#### 2.5.1. Cyclic Voltammetry

In the electrochemical cell, ultrapure water (9.5 mL), PBS (0.5 mL, 0.01 mol L^−1^), and RT (250.0 µL, 0.6 mmol L^−1^) were added sequentially. Then, after an equilibration time of 3 s, cyclic voltammograms (scan rate of 50 mV s^−1^) were recorded while the potential was scanned from −0.3 to 0.6 V. Each voltammogram was repeated 3 times.

#### 2.5.2. Square Wave-Stripping Voltammetry

In the electrochemical cell, ultrapure water (9.5 mL), PBS (0.2 mL, 0.01 mol L^−1^), and RT (10.0–100.0 µL, 0.30 mmol L^−1^) were added sequentially, and the mixture was deposited for 30 s at 0.0 V. After an equilibration time of 3.0 s, the potential was scanned from −0.3 to 0.5 V using a square wave with a frequency of 15 Hz and pulse amplitude of 500 mV. Each voltammogram was repeated 3 times. The calibration curves for RT were developed between 0.60 and 30.0 µmol L^−1^. The detection limits were calculated using the standard error (3σ) and the slope of the calibration curve. In the real sample, the standard addition method was used to eliminate matrix effects. All data were obtained at room temperature (~25 °C).

#### 2.5.3. EIS

EIS measurements were carried out at the open-circuit potential using a perturbation amplitude of 10 mV and a frequency range between 10.0 kHz and 0.10 Hz. The working electrodes were an SPC electrode, CS/SPC, and IL-CS/SPC. The tested electrolyte was K_4_Fe(CN)_6_ 10.0 mmol L^−1^ in KCl 10.0 mmol L^−1^.

## 3. Results and Discussion

### 3.1. Characterization of CS/SPC Electrode Surface with SEM

**Figure 1:**
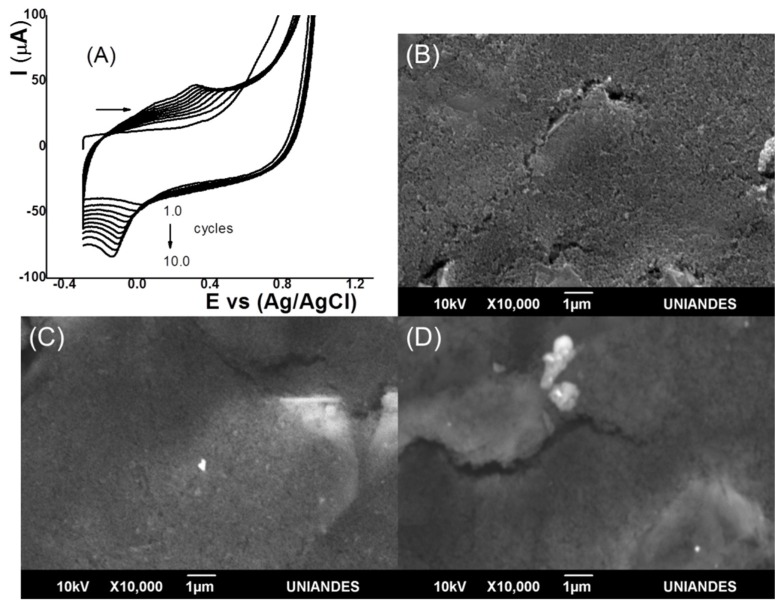
(**A**) Preparation of electromodified screen-printed carbon (SPC) electrode by cyclic voltammogram (CV) (10 cycles) and scanning electron microscopy (SEM) images of the (**B**) SPC, (**C**) chitosan (CS)/SPC electrodes, and (**D**) ionic liquid (IL)-CS/SPC.

The IL-SC composite was electrodeposited between −0.3 and 1.0 V at a scan rate of 0.05 V s^−1^ for 10 cycles. Figure 1A shows the voltammograms. An anodic peak current and a cathodic peak current were clearly observed between 0.2 and 0.4 V and −0.1 and −0.2 V, respectively, assigned to a quasireversible system of the imidazolium group of the ionic liquid [55]. When the study was done only with CS and in the absence of ILs, this redox pair was not observed. This increase in the current in each cycle indicated the presence of an IL-CS composite electrodeposited film on the electrode surface. Moreover, after more than 10 cycles, the current was not increased. The changes on the modified electrodes surfaces were investigated by SEM. Figure 1B shows the surface of the SPC electrode without CS, and IL-CS composite where a nonuniform and porous surface was observed. Meanwhile, for the CS/SPC electrode (Figure 1C), a more homogeneous and smooth surface was observed. On the other hand, the surface with the IL-CS composite (Figure 1D) showed few changes, indicating a good interaction between CS and ILs.

### 3.2. Raman Spectroscopy

Raman analysis showed that the experiment conducted at 638 nm provided the best Raman dispersion information. For the SPC electrode, only the D band (1350 cm^−1^) and the G band (1591 cm^−1^) were observed, both of which are the characteristic bands of materials with carbonaceous structures [56]. The G band is related to the energy of *sp*^2^ bonding and is assigned to normal graphitic structures. In addition, when CS was deposited on the electrode, two bands were observed at 1341 and 1589 cm^−1^, corresponding to the amino and acetamido groups, respectively [57]. In the electrode containing the IL-CS electrocomposite, the CS bands were observed to shift to higher wavenumbers, indicating that there was an interaction between the CS and ILs. The figures are shown as supporting information (Figures S1–S3)

### 3.3. Characterization of SPC, CS/SPC and IL-CS/SPC Electrodes by CV and EIS

**Figure 2:**
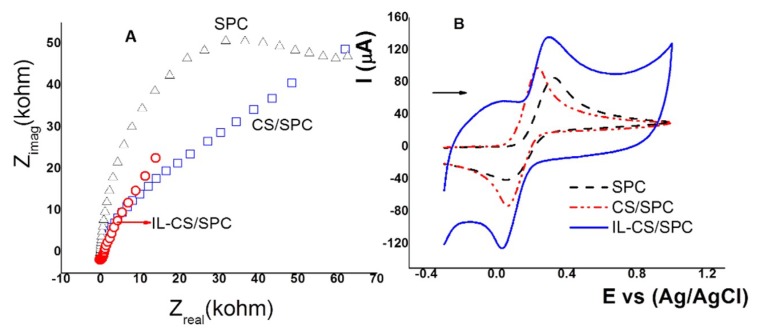
(**A**) Nyquist plot for Fe(CN)_6_^3−/4−^ 10.0 mmol L^−1^ in KCl 10 mmol L^−1^ using SPC (black curve), CS/SPC (blue curve) and IL-CS/SPC (red curve). (**B**) Cyclic voltammograms of Fe(CN) ^3−/4−^ 10.0 mmol L^−1^ in KCl 10 mmol L^−1^ using SPC (dash curve), CS/SPC (dash-dot curves) and IL-CS/SPC (solid curve). Scan rate (v) 0.1 V s^−1^.

SPC-modified electrodes were studied with K_4_Fe(CN)_6_ 10.0 mmol L^−1^ in KCl 10.0 mmol L^−1^ as test solutions to identify the conductive properties of the surface using CV and EIS. The results are shown in Figure 2. Electrochemical impedance analysis is reported in the Nyquist plot for an electrical circuit at high and low frequencies. The results of the EIS analysis for K_4_Fe(CN)_6_ using SPC, CS/SPC, and IL-CS/SPC electrodes are shown in Figure 2A. The electrochemical impedance for the SPC electrode exhibits a straight line with a slope to 90° at low frequencies that is associated with a porous surface and semi-infinite diffusion [58]. The impedance of a simple faradic reaction without diffusion can be calculated in terms of a CPE, CS/CPE, and IL-CS/CPE as

(1) $$Z_{\left(\omega \right)}=R_{s}+\frac{R_{s}}{1+{\left(j\omega \right)}^{a}Q\;R_{ct}}$$

were *Q* and *a* are the CPE parameters, *ω* is the angular frequency. *Q* represents the differential capacity of the interface in the case where *a* = 1 [59].

The charge transfer resistance (Rct) value is 35,500 Ohm. These results indicate a limitation of the faradaic process for the SPC electrode. Similar results for Rct were reported for a system with unmodified SPC electrodes [6]. The SPC electrode coated with CS and the IL-CS composite showed very different response results, first exhibiting a line with a slope close to 45° at low frequencies that can be associated with diffusion on a homogenous film. On the other hand, the charge transfer resistance (Rct) values were 4500 and 1800 Ohm. These results are related to the SEM results, where it was observed that the surfaces were more homogenous for the electrodes with CS and IL-CS composite. These results imply that IL-CS/SPC electrocomposite film facilitated the electron transfer.

Cyclic voltammograms for the K_4_Fe(CN)_6_ solution with KCl using SPC, CS/SPC, and IL-CS/SPC electrodes are shown in Figure 2B. For the SPC electrode, the anodic and cathodic peak currents were 63.8 and −35.8 µA with ∆V of 0.22 V for a quasireversible reaction. When SPC electrode was coated with CS and IL-CS electrocomposite, anodic and cathodic peak currents were ±70.0 µA, indicating that the amount of oxidized Fe^2+^ is the same as the amount of Fe^3+^, allowing a reversible redox reaction. On the other hand, the observed charges for SPC, CS/SPC, and IL-CS/SPC electrodes were 0.132, 0.140 and 0.167 mC, respectively. These results are similar to those observed by EIS, where the presence of CS and ILs improves the conductivity of the surface of the SPC electrode. Moreover, the background current of the IL-CS/SPC electrode is higher than that of the large surface area of the composite film. For the electrodes modified with CS, ILs, and nanoparticles, a more considerable increase in the values of the anodic and cathodic current was not observed [54].

### 3.4. Activity of RT Using SPC, CS/SPC and IL-CS/SPC Electrodes

**Figure 3:**
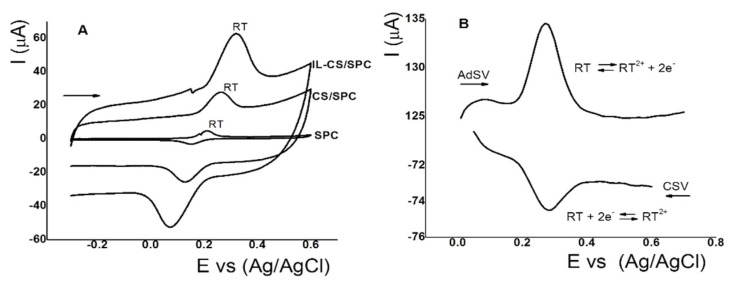
(**A**) Cyclic voltammograms of rutin (RT) (35.0 mmol L^−1^) pH 2.5 (PBS) using SPC, CS/SPC, and IL-CS/SPC electrodes. 0.1 V s^−1^. (**B**) cathodic and adsorptive stripping voltammograms of RT (3.5 µmol L^−1^). Conditions: pH 2.5 (PBS); cathodic stripping voltammetry (CSV): Eads 0.6 V; t_ads_ 60 s; adsorptive stripping voltammetry (AdSV): E_ads_ 0.1 V; t_ads_ 60 s. Other conditions: Step amplitude 0.01 V; pulse amplitude 0.05 mV; and frequency 15 Hz.

Electroactivity properties for RT are related to the ortho-dihydroxy-phenol group in the chemical structure where two proton–electron pairs can be oxidized between 0.0 and 0.5 V [60]. Figure 3A shows the cyclic voltammograms for RT (35.0 µmol L^−1^) at 0.10 V s^−1^ in pH 2.5 with PBS, using SPC, CS/SPC, and IL-CS/SPC electrodes. The results clearly show that RT shows activity on the surface of the three electrodes through a quasireversible reaction. The ∆E values for RT using SPC, CS/SPC, and IL-CS/SPC were 0.06, 0.14, and 0.23 V, respectively. These results indicate that more energy is required to oxidize RT with the modified electrode. This may be due to the width of the film. On the other hand, the anodic peak currents for RT with SPC, CS/SPC, and IL-CS/SPC electrodes were 3.95, 11.0, and 29.1 µA, respectively. These results showed that the current increased by more than 100% with IL-CS/SPC, indicating that the concentration of RT on the surface of the IL-CS/SPC electrode is higher. It is possible that the positive charge of the CS as NH^3+^ in acidic medium interacts with the imidazolium anion. Therefore, the CS–IL composite acts as an extraction agent for RT through an affinity interaction [47]. Similar results have been reported for other analytes such as for the interaction of dopamine with ILs [61]. With respect to the potential peak for RT, it was reported at more positive values with ILs on carbon paste [33] and CS on glassy carbon [42]. To select the most sensitive technique, cathodic stripping voltammetry (CSV), the most popular stripping voltammetric technique suitable for the determination of organic substances, and adsorptive stripping voltammetry (AdSV) that shows higher sensitivity due to the adequate accumulation potential value, were evaluated for RT oxidation. In CSV and AdSV (Figure 3B) using square wave, the anodic and cathodic peak potentials of RT (3.5 µmol L^−1^) are observed at 0.28 V with the cathodic and anodic peak currents of −2.36 and 3.98 µA, respectively. These results showed that AdSV exhibit a higher response. Therefore, it has higher sensitivity for RT analysis. AdSV is chosen as the optimal technique for subsequent studies.

### 3.5. Influence of the Scan Rate of RT on the IL-CS/SPC Electrode

**Figure 4:**
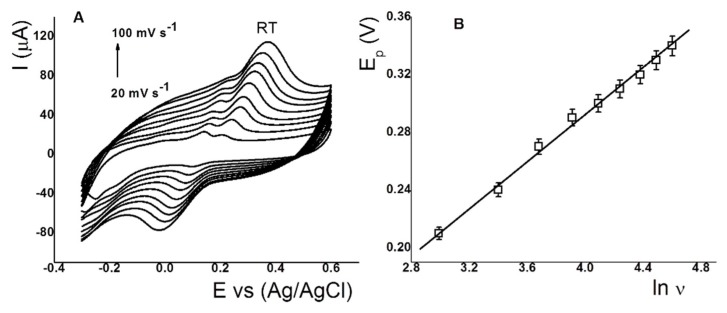
(**A**) Voltammograms and (**B**) plots of the dependence of anodic peak potentials vs. ln ν for RT (35.0 µmol L^−1^) using IL-CS/SPC electrode.

The mass-transfer process for RT on the IL-CS/SPC electrode was studied to elucidate the influence of the scan rate (ν) on the anodic peak current at pH 2.5 (Figure 4A). Anodic peak currents were studied at the rates between 0.02 and 0.10 V s^−1^. A linear trend was observed at low and high scan rates. The regression equation was ip_a_ (µA) = −4.549 + 0.439ν (correlation coefficient R^2^ = 0.995). This result indicates that the process on the IL-CS/SPC electrode is adsorption-controlled. Moreover, the plots of the dependence of the anodic peak potentials vs. ln ν for RT are fitted with the regression equation of Ep_a_ (V) = −0.033 + 0.069 ln ν (correlation coefficient R^2^ = 0.990). This slope value is close to the value of 0.059 in the Nernst equation. This linear relationship indicates that the electro-oxidation of RT shows a stoichiometry of 2H^+^:2e^−^. Similar results were reported for RT using Cu–CS/MWCNT carbon nanotubes [42] and ionic liquid-modified carbon-paste electrodes [33].

### 3.6. Effect of pH on the Anodic Peak Current of RT on the IL-CS/SPC Electrode

**Figure 5:**
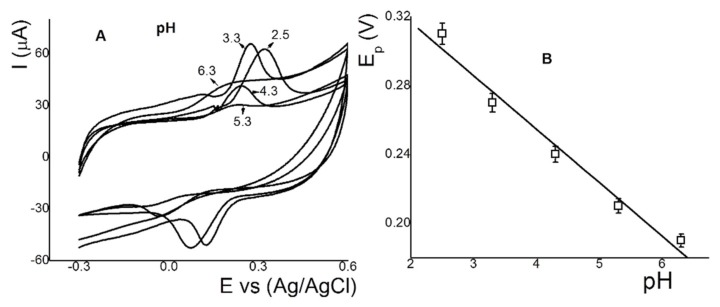
(**A**) Voltammograms of the effect of the pH (6.3, 5.3, 4.3, 3.3, and 2.5) for 35.0 μmol L^−1^ of RT, and (**B**) plots of the dependence of anodic peak potentials vs. pH for RT using IL-CS/SPC. Conditions: scan rate of 0.1 V s^−1^.

Adsorptive properties depend largely on the pH value in the electrochemical cell. The pH influence on anodic peak current for RT was studied at pH range 2.5–6.3 using PBS (0.001 mol L^−1^) with 35.0 µmol L^−1^ of RT (0.1 V s^−1^) (Figure 5A). Anodic peak potentials for RT were shifted slightly toward more negative values with increasing pH, suggesting that protons participate directly in the oxidation of RT. The anodic peak current maximum was obtained at pH 2.5 and this pH was used for further experiments. Moreover, the plots of the dependence of the anodic peak potentials on pH for RT were fitted with the regression equation Epa (V) = 0.368 + 0.049pH (correlation coefficient R^2^ = 0.992). This value of the slope value is close to the value of 0.059 in the Nernst equation. This linear relationship indicates that the electro-oxidation for a quasireversible reaction of RT shows the stoichiometry of 2H^+^:2e^−^. These results are the same as those described in Section 3.3 and as previously reported in the literature [62].

### 3.7. Influence of the Adsorption Time (t_ADS_), Adsorption Potential (E_ADS_) and SWV Parameters

To optimize the adsorption of RT on the IL-CS/CPE surface, t_ADS_, E_ADS_, and SWV parameters were studied with an RT concentration of 3.5 µmol L^−1^. The results showed that with an increase in the adsorption time, the anodic peak current increased to 60 s, with no increase observed for longer time. This may be due to the saturation of the electrode surface. 60 s is chosen as the optimal t_ADS_ for subsequent studies. E_ADS_ was studied between −0.2 and 0.2 V. The results showed that anodic peak current for RT was higher at the positive potential values. 0.1 V was chosen as the optimal potential value. This potential value it is not high enough to oxidize RT. Therefore, RT was adsorbed on the IL-CS/SPC electrode surface. The effects of the square wave parameters, such as the pulse amplitude (V) and frequency (Hz), on the anodic peak current were also studied in the ranges of 0.01–0.1 V and 5.0–30.0 Hz, respectively. The anodic peak current for RT increased with the increase in the pulse amplitude and frequency to 0.09 V and 10.0 Hz, respectively. Therefore, these values were chosen as optimal for the subsequent studies.

### 3.8. Analytical Parameters, Stability and Repeatability

**Figure 6:**
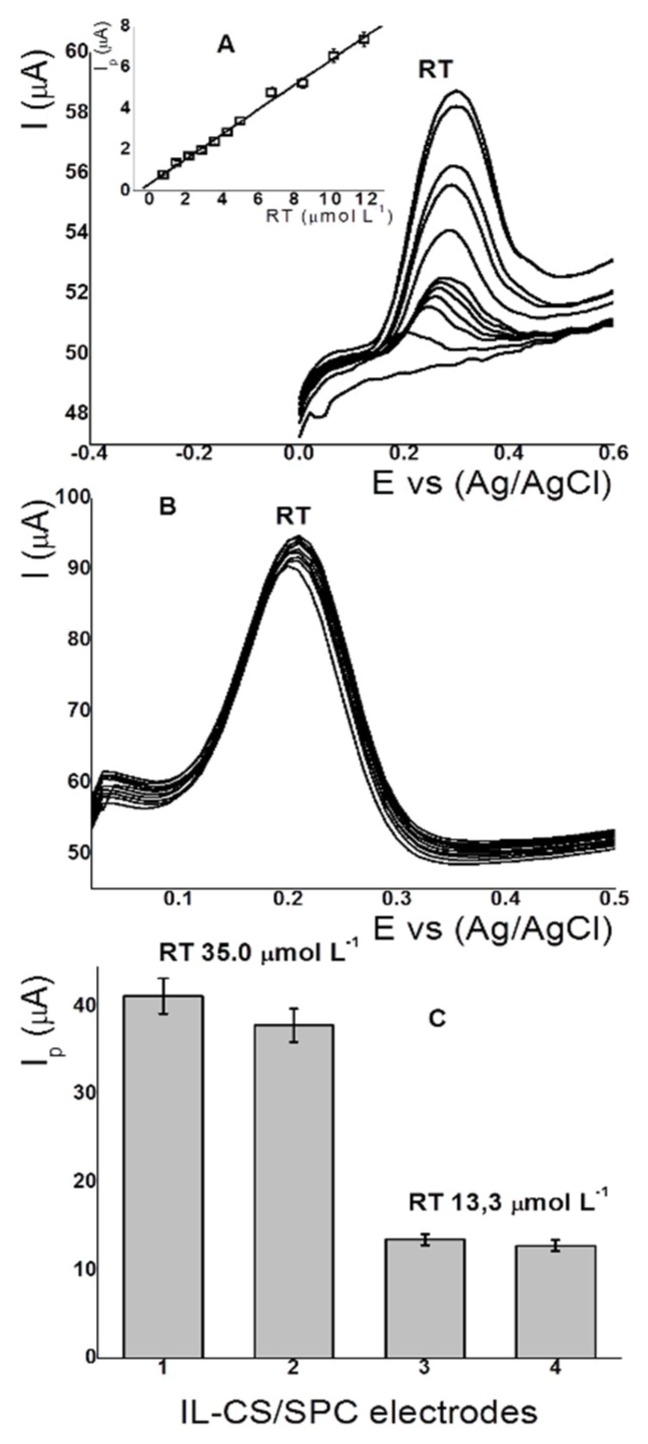
(**A**) Adsorptive voltammograms and calibration curve (insert) of RT between 0.75−12.5 µmol L^−1^; (**B**) adsorptive voltammograms (n = 10) for RT 35.0 µmol L^−1^; (**C**) and anodic peak current for RT 35.0 and 13.3 µmol L^−1^ using four different CS/SPC electrodes. Conditions: pH 2.5; E_ADS_ 0.1 V; t_ADS_ 60 s.

Figure 6 shows the voltammograms of the calibration curve (calibration-curve insert), stability, and reproducibility of the IL-SC/SPC electrode. Detection limits was calculated from the calibration curve obtained between 0.75 and 12.5 µmol L^−1^ of RT with the optimized conditions: pH 2.5 (0.01 mol L^−1^ PBS), E_ADS_ 0.1 V, and t_ADS_ 60 s. The regression equation for the anodic peak currents (Figure 6A) was Ipa = 0.397 ± 0.01 + 0.596 ± 0.09C_RT_ (correlation coefficient R^2^ = 0.997). The DL obtained for RT based on the values of the slopes and the random errors in x and y was 0.07 µmol L^−1^. The stability and durability of the sensor was evaluated by performing SWAdV after 10 consecutive measurements for a RT concentration of 35.0 µmol L^−1^. The results are shown in Figure 6B. Anodic peak currents for RT increased by only approximately 5.0%. This corresponds to the increase of 0.5% in the current (μA) for each measurement. Moreover, the relative standard deviation (RSD) was 0.86% (n = 10). The reproducibility was 1.5% (n = 7) for RT (35.0 and 13 µmol L^−1^) using four different electrodes (Figure 6C). These results indicate that the performance of the sensor allows it to be used for a long period of time without an appreciable reduction in its activity. The proposed method using IL-CS/CPE for RT detection was compared to other modified electrodes, as summarized in Table 1.

### 3.9. Interference, Validation and Study

**Figure 7:**
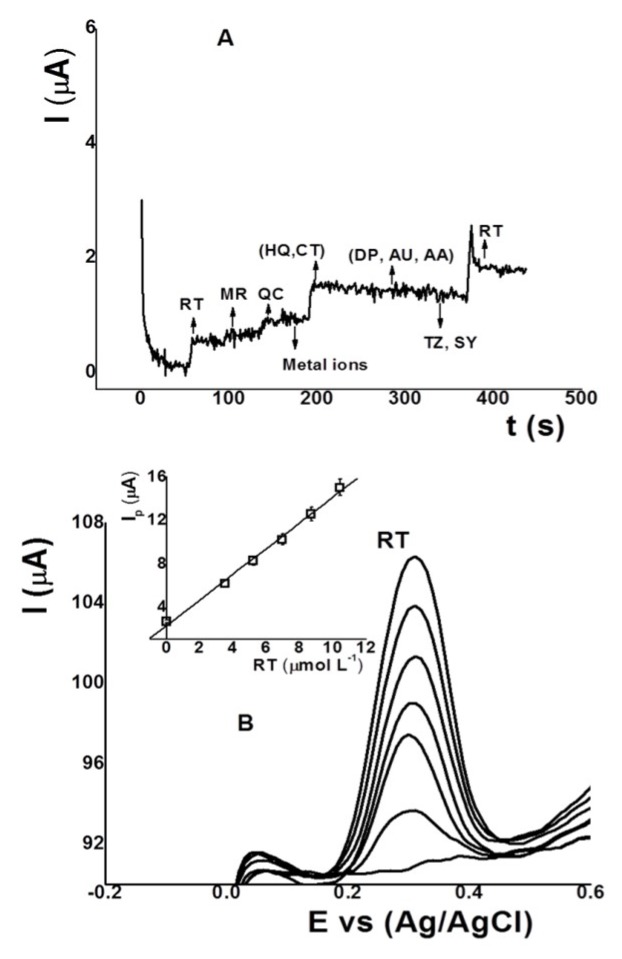
(**A**) Amperograms for RT with morin (MR), quercetin (QC), metal ions, hydroquinone (HQ), catechol (CT), dopamine (DP), ascorbic acid (AA), uric acid (UA), tartrazine (TZ), sunset yellow (SY) and (**B**) adsorptive voltammograms and calibration curves (inset) of sample 1 * (Table 2). Using the IL-CS/SPC electrode under the same conditions as Figure 6.

The possible interfering substances in the detection of RT with the IL-CS/SPC electrode were evaluated by the amperometric technique at 0.3 V with metal ions using an ICP multielement standard solution IX (Merck) containing As, Be, Cd, Cr(VI), Hg, Ni, Pb, Se, and Tl 100 mg L^−1^, hydroquinone (HQ), catechol (CT), uric acid (UA), ascorbic acid (AA), dopamine (DP), dye colors such as tartrazine (TZ) and sunset yellow (SY), and others flavonoids such as quercetin (QC) and morin (MR) in concentrations ten times higher. The results showed that only HQ and CT can cause interference with the signal for RT. Figure 7A shows the amperograms for RT in the presence of the substances mentioned above. These results suggest that the IL-SC/SPC electrode can be used for the analysis of natural samples and foods.

The accuracy for RT detection using the IL-CS/SPC electrode was evaluated with the standard control urine chemistry from Bio-Rad spiked with known amounts of RT. The results are summarized in Table 2. The relative error of the four samples were close to 10.0%, indicating acceptable accuracy, considering the concentration of smaller than 5.0 µmol L^−1^. Moreover, the slopes of the calibration curves for RT were 1.18, 1.09, and 0.90. The small differences in the slope values indicate that the matrix of the sample did not affect the activity of the sensor. The voltammograms for sample 1 of Table 2 are shown in Figure 7A.

### 3.10. Analytical Application

An IL-CS/SPC electrode was used in the detection and quantification of RT in tropical fruits such as orange, lemon, and agraz (*Vaccinium meridionale* Swartz) obtained at a supermarket in Ibagué, Colombia. The concentrations of RT were calculated by standard addition. Each sample was analyzed at pH 2.5 with PBS. The results are shown in Table 3. These results were similar to a previous report where the concentration of RT was found to be higher in lemon than in orange [42]. Moreover, the amount of RT detected in agraz was higher and was similar to the amount of RT detected in grapefruit by capillary electrophoresis [63] and electroanalytical method [64].

## 4. Conclusions

The IL-CS composite deposited onto SPC electrode showed high activity and selectivity towards RT oxidation. Moreover, it allowed the detection and quantification in natural samples without any pretreatment. Additionally, the sensor was shown to be stable, reproducible, sensitive, and easily developed.

## Figures and Tables

**Table 1:** Modified electrodes for rutin determination.

Electrode	Materials	DL (µmol L^−1^)	Application	Reference
GCE	Cu–CS/MWCNT	0.01	Fruits	[42]
ABPE	CS/ MWCNT	0.01	Pharmaceutical samples	[43]
CPE	IL	0.01	Pharmaceutical samples	[33]
GCE	CS/G	0.50	Pharmaceutical samples	[44]
GCE	IL-MWCNT	0.02		[47]
SPC	CS	0.09	Tea	[63]
SPC	IL-CS	0.07	Tropical fruits	This work

GCE: glassy carbon electrode; CPE: carbon paste electrode; ABPE: acetylene black paste electrode; Cu–CS/MWCNT: copper-complexed chitosan/multiwalled carbon nanotubes; G-CS-polyAA: graphene nanosheets, chitosan, and a poly (amido amine).

**Table 2:** Results for RT in urine chemistry control standard.

Sample	Added (µmol L^−1^)	Found (µmol L^−1^)	% Relative error
1 *	1.74	1.94 ± 0.01	11.5
2	1.74	1.95 ± 0.02	12.1
3	3.50	3.92 ± 0.05	12.0
4	3.50	3.72 ± 0.04	6.3

* voltammograms are shown in Figure 7B (insert calibration curve).

**Table 3:** Results for RT in tropical fruit samples.

Sample	Found (mol µL^−1^)	RDS (%)
	RT	RT
Agraz extract	18.3	0.50
Orange	2.30	0.05
Lemon	4.20	0.08

## Supplementary Materials

The following are available online at http://www.mdpi.com/1424-8220/18/9/2934/s1, Raman spectroscopy of SPC, CS/SPC and IL-CS/SPC. Were performed in a RIBA Yovin-Ivon spectrometer (Kyoto, Japan) using various laser wavelengths (532, 638, and 786 nm).
