# Supplementary material for: Electrocomposite Developed with Chitosan and Ionic Liquids Using Screen-Printed Carbon Electrodes Useful to Detect Rutin in Tropical Fruits
Source: Sensors (Basel). 2018 Sep 4;18(9):2934. doi: 10.3390/s18092934 (PMC6164375; doi:10.3390/s18092934)
Supplement: Supplementary file 1 [file sensors-18-02934-s001.pdf]

Supporting Information

Article

Electrocomposite Developed with Chitosan and Ionic Liquids Using Screen-Printed Carbon Electrodes Useful to Detect Rutin in Tropical Fruits

Lili Muñoz [1], Verónica Arancibia [2], Olimpo García-Beltrán [3], Edgar Nagles [3,*], John J. Hurtado [4]

1 Ingeniería Agroindustrial, Facultad de Ingeniería Agronómica, Universidad del Tolima, Calle 67 No. 53−108 B, Ibagué-Tolima,730001 Colombia; lilifasulimondragon@gmail.com
2 Facultad de Química, Pontificia Universidad Católica de Chile, Vicuña Mackenna 4860, Santiago 7820436, Chile; darancim@uc.cl
3 Facultad de Ciencias Naturales y Matemáticas, Universidad de Ibagué, Carrera 22 Calle 67, Ibagué 730001, Colombia; jose.garcia@unibague.edu.co
4 Departamento de Química, Universidad de los Andes, Carrera 1 No. 18A−12, Bogotá 111711, Colombia; jj.hurtado@uniandes.edu.co
* Correspondence: edgar.nagles@unibague.edu.co; tel.: +57-8270-9444.

Received: 9 August 2018; accepted: 28 August 2018; published: date

Overview
Supplementary figures

Materials and methods

-Raman spectroscopy was performed in a RIBA Yovin-Ivon spectrometer using various laser wavelengths (532, 638, and 786 nm)

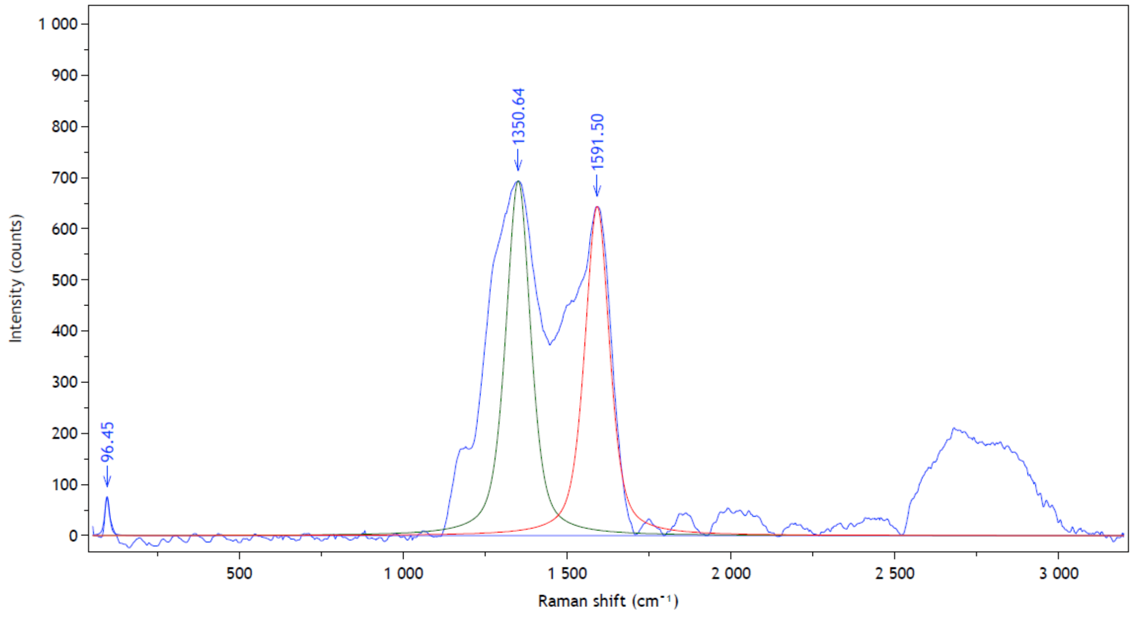

Figure S1. RAMAN spectrum of electro SPC.

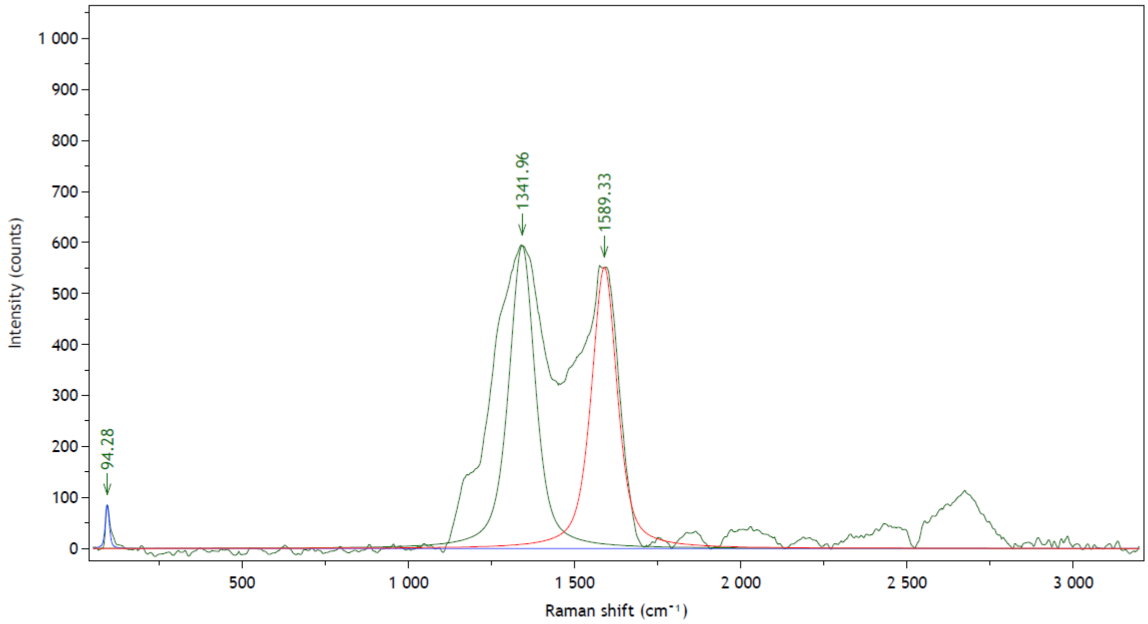

Figure S2. RAMAN spectrum of electrode with CS/SPC.

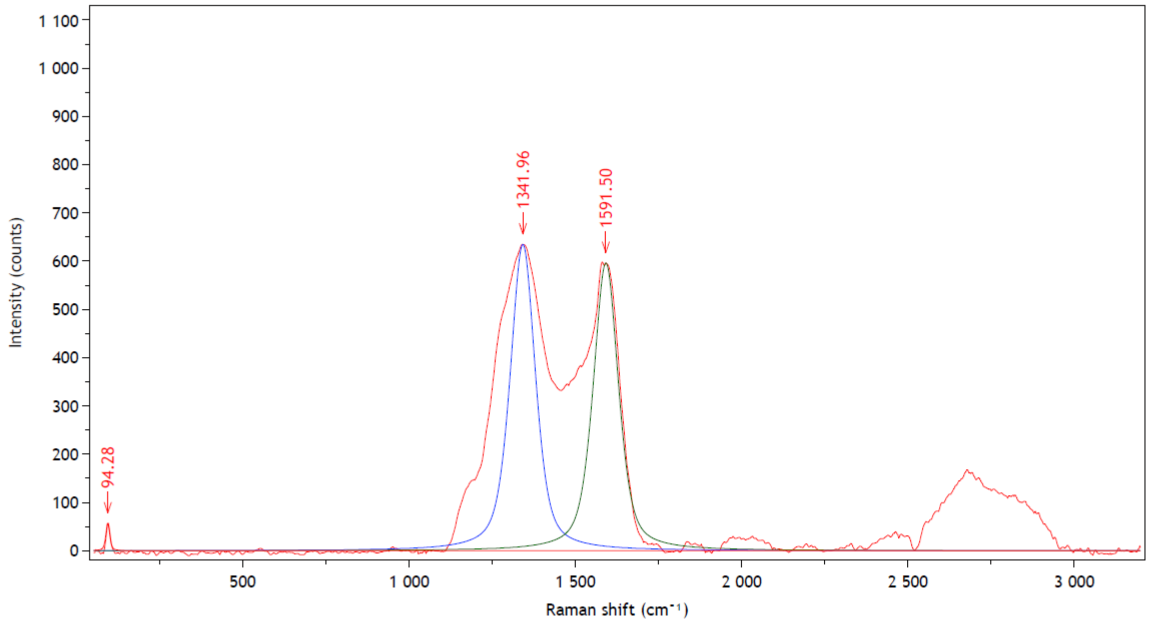

Figure S3. RAMAN spectrum of electrode with IL-CS/SPC.
